# Pediatric Cerebral Spetzler-Martin Grade 5 Arteriovenous Malformation

**DOI:** 10.7759/cureus.25972

**Published:** 2022-06-15

**Authors:** Allen Mao, Nausheen Khuddus, Hoang D Duong

**Affiliations:** 1 Diagnostic Radiology, HCA Florida North Florida Hospital, Gainesville, USA; 2 Pediatric Ophthalmology, HCA Florida North Florida Hospital, Gainesville, USA; 3 Interventional Neuroradiology, HCA Florida North Florida Hospital, Gainesville, USA

**Keywords:** arteriovenous malformations, pediatric, sm5, spetzler-martin, avm

## Abstract

Brain arteriovenous malformations (AVMs) are a type of intracranial high-flow vascular malformation composed of enlarged feeding arteries and draining veins. Without a capillary bed connection, there can be damage to the walls of the arteries and veins, which causes abnormally high blood flow. AVMs are rarely found in children and are thought to expand over time until they become symptomatic. We present an interesting case of a pediatric male who initially presented with seizure-like episodes and was found to have a large frontoparietal Spetzler-Martin (SM) grade 5 AVM after cerebral digital subtraction angiography. Unfortunately, given how much eloquent brain and deep cortical structures were intertwined in the SM5 AVM, risk-benefit analysis favored observation over surgical management. The patient’s clinical presentation and imaging findings are described followed by a discussion of the epidemiology, grading system, and treatment of AVMs. After extensive literature review, this clinical entity has been previously reported but is relatively rare in children with prognosis and therapy correlating to the severity of the SM index.

## Introduction

Brain arteriovenous malformations (AVMs) are a type of intracranial high-flow vascular malformation composed of enlarged feeding arteries and draining veins. Without a capillary bed connection, there can be damage to the walls of the arteries and veins, which causes abnormally high blood flow. AVMs are rarely found in children and are thought to expand over time until they become symptomatic. There is a 0.02% prevalence in children and no gender predilection [1]. They can present in various ways including an incidental finding in asymptomatic patients, seizure, stroke, or even hemorrhage which is the most feared event. AVMs have a higher rate of rupture than children because they usually are detected only after they rupture [2]. The differential diagnosis for AVMs can include intracranial hemorrhage, migraine headache, dissection syndromes, cerebral artery stroke, and cerebral venous thrombosis [3].

We highlight the hospital course of a pediatric patient who presented with seizure-like episodes secondary to a large frontoparietal cerebral arteriovenous malformation. The patient’s clinical presentation and imaging findings are described followed by a discussion of the epidemiology, grading system, and treatment of AVMs. The Spetzler-Martin (SM) grading scheme is used in order to characterize the AVM, which can be treated via microsurgical resection, endovascular occlusion/embolization, stereotactic radiosurgery (SRS), or observation.

## Case presentation

Our patient is a 15-year-old male with a past medical history of asthma, scoliosis, and strabismus and is status-post surgical correction in 2008. The patient presented to the emergency department (ED) for dysarthria, right arm weakness, and headache after playing video games. The patient’s mother stated that her son often clenches his jaw as he gets intense with video games. The patient complained to his mom that “his right arm was not working and felt weak,” which lasted for 15 minutes. In addition, the mother thought his speech was slurred. By the time the patient arrived at the ED, he was at his baseline. Of note, the patient had a similar episode two years prior with right arm weakness and slurred speech while playing video games, but it was thought to be a one-time episode and secondary to overexertion as CT findings were negative.

**Figure 1:**
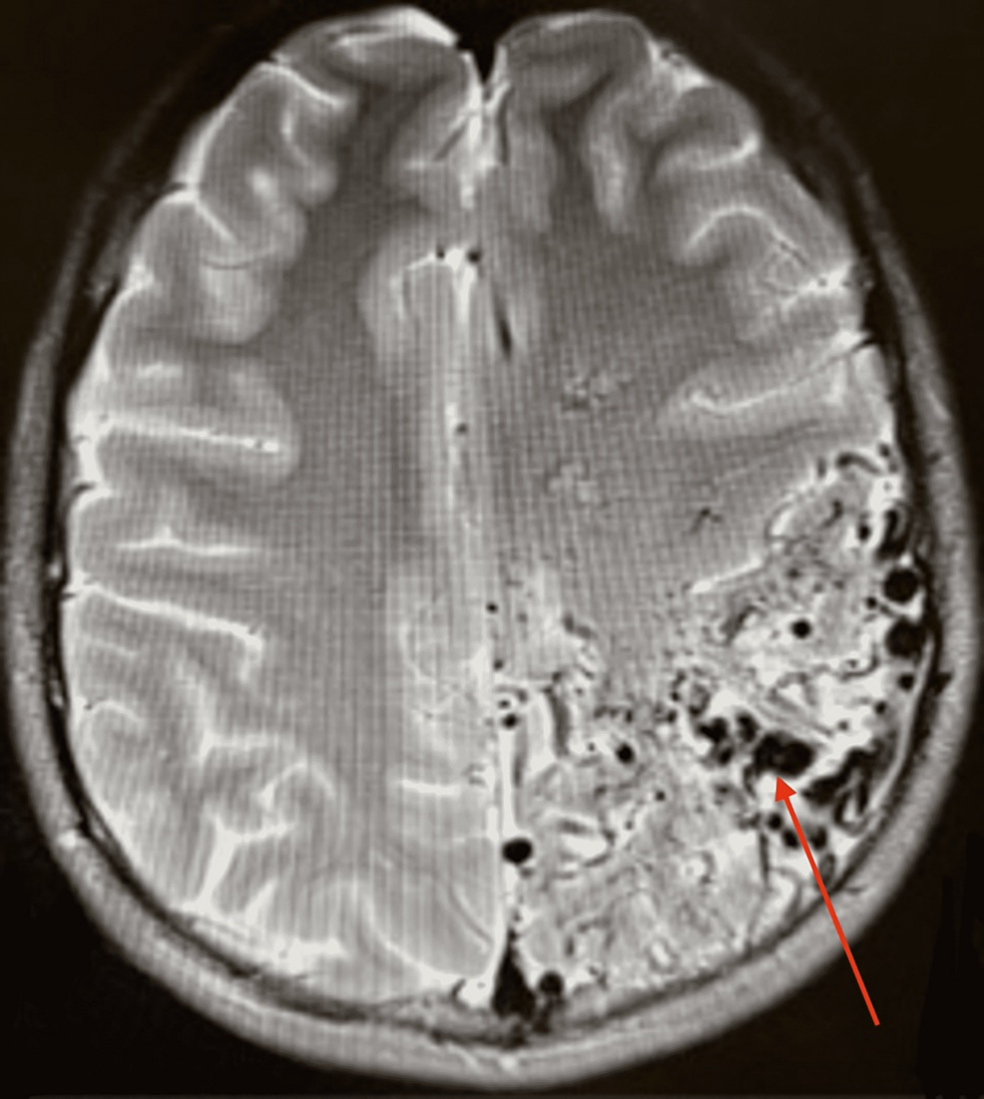
T2-weighted MRI This figure shows an AVM in the frontoparietal region with several flow voids and a characteristic “bag of worms” appearance (red arrow).

**Figure 2:**
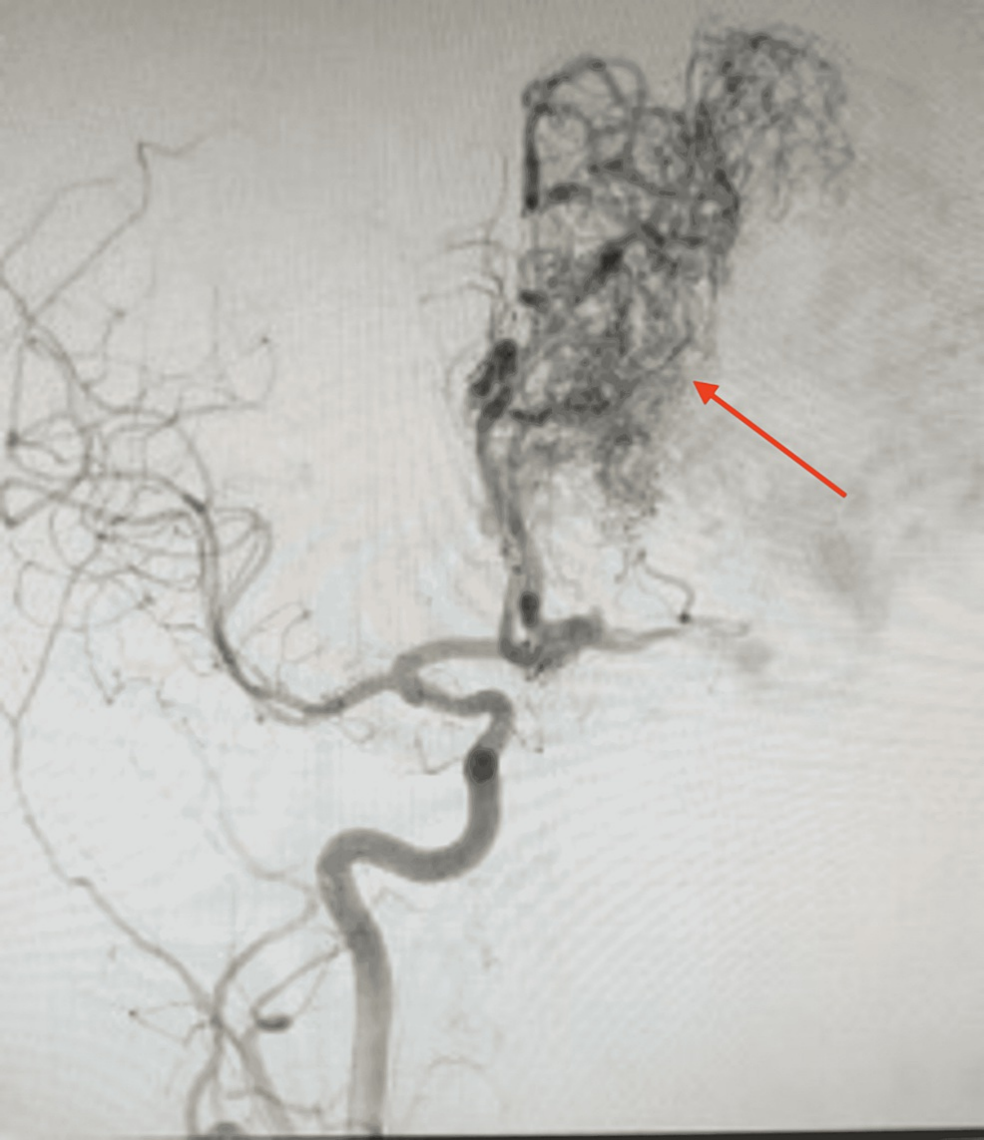
Cerebral digital subtraction angiogram (arterial phase) Right transradial cerebral digital subtraction angiogram (arterial phase) shows a large Spetzler-Martin grade 5 (SM5) left frontoparietal AVM measuring 8.5 cm x 8.5 cm x 7.5 cm (red arrow). AVM: Arteriovenous malformation.

**Figure 3:**
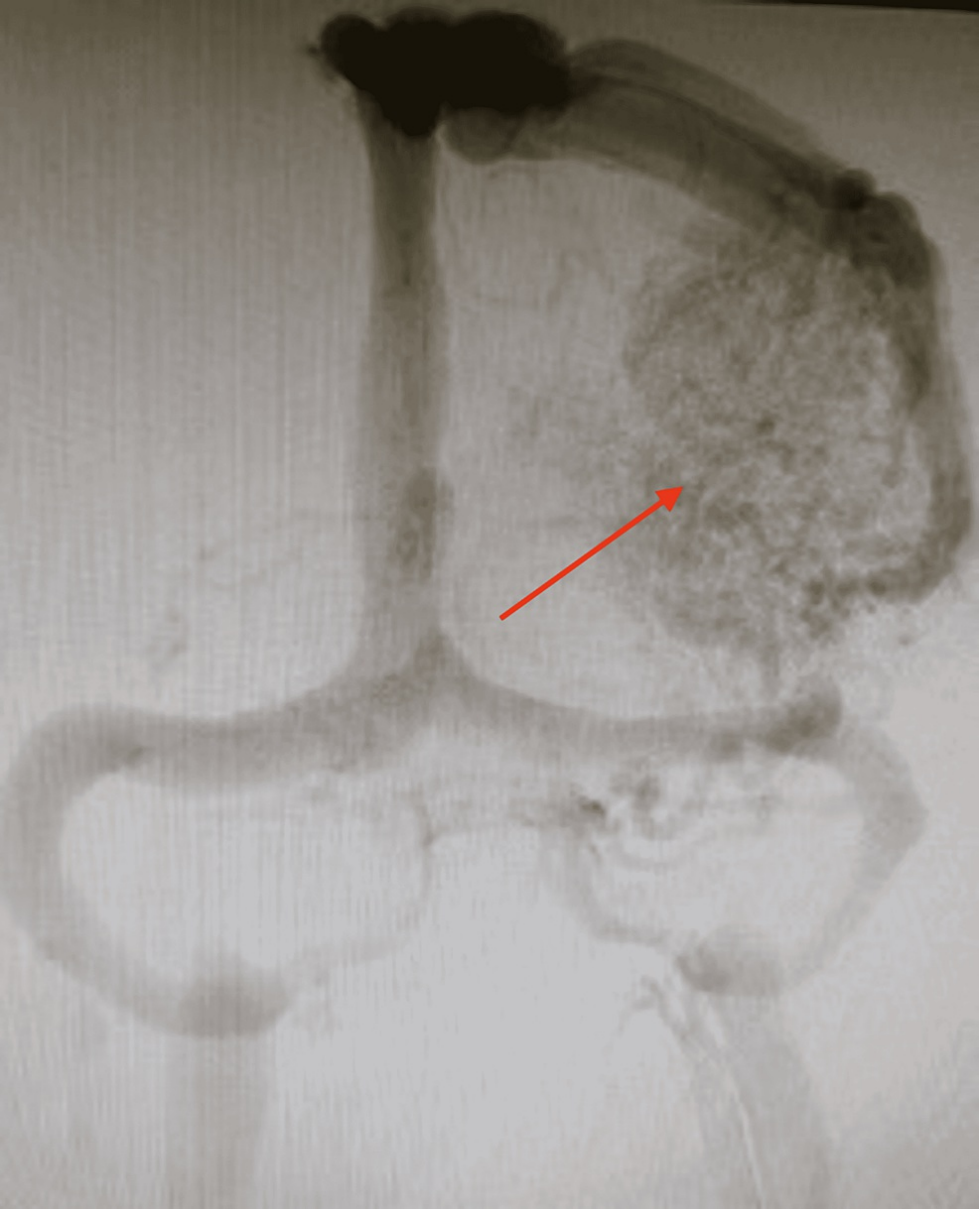
Cerebral digital subtraction angiogram (venous phase) Right transradial cerebral digital subtraction angiogram (venous phase) shows a large Spetzler-Martin grade 5 (SM5) left frontoparietal AVM measuring 8.5 cm x 8.5 cm x 7.5 cm (red arrow). AVM: Arteriovenous malformation.

Radiographic imaging was again performed in the ED. Non-contrast CT scan showed no signs of acute/subacute ischemia, few dystrophic calcifications, and a small 5-mm midline shift to the right. T2-weighted MRI showed an AVM in the frontoparietal region with several flow voids and a characteristic “bag of worms” appearance (Figure 1). The diagnosis was confirmed by interventional neuroradiology who performed a right transradial cerebral digital subtraction angiogram showing a large Spetzler-Martin grade 5 (SM5) left frontoparietal AVM measuring 8.5 cm x 8.5 cm x 7.5 cm (Figures 2, 3). The AVM was fed by the left middle cerebral artery (MCA) and left anterior cerebral artery (ACA) feeders with deep and superficial venous drainage with large cortical veins. There was also the involvement of eloquent structures including basal ganglia, insula, and central white matter. There was no evidence of intranidal aneurysm, ischemia, or associated intracranial hemorrhage.

Neurology and neurosurgery were consulted for treatment recommendations, and they determined that the AVM had likely been there for much or all of the patient’s life since birth. His two previous symptomatic episodes were likely secondary to seizures due to hemosiderin-mediated irritation of the cortex by the AVM. The patient was started on Keppra for seizure management to prevent future episodes. Unfortunately, given how much eloquent brain and deep cortical structures were intertwined with this cerebral AVM, it would be devastating neurologically to excise surgically, and as such, risk-benefit did not favor surgery. The patient has future follow-up appointments with endovascular and radiosurgery physicians to discuss whether risk-benefit would favor intervention using those modalities. In the meantime, the patient should avoid strenuous physical activity and significant dehydration to avoid the feared complication of AVM rupture causing intracranial hemorrhage. The constellation of clinical symptoms and MRI/cerebral angiogram radiographic findings led to the diagnosis of a large frontoparietal SM5 AVM that presented as seizures.

## Discussion

Arteriovenous malformations are thought to represent a congenital abnormality but are rarely found incidentally in the very young and are thought to expand over time. Their origin is uncertain but thought to be multifactorial. Their development may involve dysregulation of vascular endothelium growth factor (VEGF) receptor-mediated endothelial proliferation and cytokine-mediated vessel remodeling. They can present in various ways including an incidental finding in asymptomatic patients, seizure, stroke, or even hemorrhage, which is the most feared event [4]. When patients become symptomatic, AVMs can be found on imaging. The incidence of AVMs is rare in pediatric patients but carries a higher rate of rupture than their adult counterparts, which could be attributed to the detection of most pediatric AVMs only after they rupture.

Radiographic imaging is an important way to diagnose AVMs and further classify them by severity. The diagnosis is difficult on non-contrast CT, which is largely unspecific and may only show dystrophic calcifications. The gold standard is digital subtraction angiography (DSA), which can accurately delineate the location of the AVM. After contrast administration, the pattern of venous drainage and the number of feeding vessels can be determined. On DSA, an AVM appears as a tightly packed mass of enlarged feeding arteries that have a “bag of worms” appearance, which supplies a central nidus. T2-weighed MRI is another imaging modality that will show flow voids. Fast flow generates flow voids, which is a low signal seen in vessels that contain vigorously flowing blood. Flow void is commonly a misnomer as it actually indicates vascular patency due to the abnormally high blood flow between the artery and veins with no intervening capillary bed to slow it down.

The SM arteriovenous malformation grading system classifies AVMs by allocating points for various angiographic features of the intracranial AVM to give a score that predicts the morbidity and mortality for perioperative risk. The grade is equal to the sum of points in three categories, for a minimum of grade I and a maximum of grade V. The size is defined by the largest diameter of the nidus on angiography and ranges from small (<3 cm) for 1 point, medium (3-6 cm) for 2 points, to large (>6 cm) for 3 points. Involvement of non-eloquent areas yields a score of 0 points, whereas eloquent areas have a score of 1 point. Eloquent areas include sensorimotor, language and visual cortex, the hypothalamus and thalamus, the internal capsule, the brain stem, the cerebellar peduncles, and the deep cerebellar nuclei. Finally, only superficial venous drainage has 0 points, and the involvement of deep venous drainage has 1 point. Superficial veins primarily drain the cerebral cortex, whereas deep veins drain the deep structures within the hemispheres. The pediatric patient described above had a maximum grade of SM5 due to 3 points given for large size (>6 cm), 1 point given for the involvement of eloquent areas, and 1 point given for deep venous drainage.

Treatment options and rate of complications are dictated in part by the SM grade. In general, the four options available are microsurgical resection, endovascular occlusion/embolization, stereotactic radiosurgery (SRS), and observation. Microsurgical resection is the first-line therapy due to its high cure rate and low risk of complications and is commonly used for SM1- and SM2-grade AVMs [5]. In endovascular embolization, a catheter deposits particles of a glue-like substance in the affected artery to block blood flow. In SRS, precisely focused and highly targeted radiation beams are deployed to destroy the AVM by damaging blood vessels. The scarred blood vessels slowly clot off in one to three years following treatment. However, this latency period between treatment and obliteration gives an opportunity for hemorrhage and other associated complications to arise. Therefore, SRS is reserved for AVMs that are in inaccessible locations or in deep eloquent areas that could be associated with postoperative neurologic deficits. For SM4- and SM5-graded AVMs, observation is recommended as the risk-benefit analysis does not favor treatment. Occasionally, AVMs have been known to spontaneously resolve. The feared complication of an untreated AVM is hemorrhage, and the annual risk is 2%-3% [6].

## Conclusions

We present an interesting case of a pediatric male with seizure-like symptoms who was found to have a frontoparietal SM5-graded AVM after cerebral DSA. Given the large size and involvement of eloquent brain structures, observation was the recommended treatment.
